# Supplementary material for: An XA21-Associated Kinase (OsSERK2) Regulates Immunity Mediated by the XA21 and XA3 Immune Receptors
Source: Mol Plant. 2014 Jan 30;7(5):874–92. doi: 10.1093/mp/ssu003 (PMC4064043; doi:10.1093/mp/ssu003)
Supplement: Supplementary Data [file supp_7_5_874__index.html]

An XA21-Associated Kinase (OsSERK2) regulates immunity mediated by the XA21 and XA3 immune receptors — An XA21-Associated Kinase (OsSERK2) Regulates Immunity Mediated by the XA21 and XA3 Immune Receptors — An XA21-Associated Kinase (OsSERK2) Regulates Immunity Mediated by the XA21 and XA3 Immune Receptors — An XA21-Associated Kinase (OsSERK2) Regulates Immunity Mediated by the XA21 and XA3 Immune Receptors — Supplementary Data 

# An XA21-Associated Kinase (OsSERK2) Regulates Immunity Mediated by the XA21 and XA3 Immune Receptors

## Supplementary Data

Data files

**Files in this Data Supplement:**

- Supplementary Data - Supplementary Data
