# Supplementary material for: An XA21-Associated Kinase (OsSERK2) Regulates Immunity Mediated by the XA21 and XA3 Immune Receptors
Source: Mol Plant. 2014 Jan 30;7(5):874–92. doi: 10.1093/mp/ssu003 (PMC4064043; doi:10.1093/mp/ssu003)
Supplement: Supplementary Data [file supp_ssu003_ssu003_Supplemental_file_Chen_et_al.pdf]

Supplemental Experimental Procedures

Transformation constructs

For RNAi vector construct design, A 383 bp unique cDNA sequence of *OsSerk2Ri* (amplified by primer pair *OsSerk2Ri-1*/*-2*: (5'-CACCATGATCCGCCGCTTGAAT-3'/5'- CCAATCGAGCAACATCACAT -3')) and a 432bp unique cDNA sequence of *OsSerk1* (amplified by primer pair *OsSerk1Ri-1*/*-2*: (5'-CACCATCCGTGCACTTGGTTTCAT -3'/5'-AAGGGTTGTTGGCAAAACTG-3')) were cloned into pENTR™/D-TOPO® (Invitrogen) vector and then inserted into pANDA (Kindly provided by Professor Ko Shimamoto, Nara Institute of Sciene and Technology, Japan) through LR recombination to create *OsSerk2Ri* and *OsSerk1Ri* constructs.

For the yeast two-hybrid assay vector construct design, the coding cDNA sequences with the stop codon of OsSERK2 variants, OsSERK2JMK and OsSERK2JK containing juxtamembrane and kinase domains (JMK), OsSERK2TJM containing partial sequence of the transmembrane, juxtamembrane and kinase domains (TJK), XA21JK, XA3JMK, OsFLS2JMK, and OsBRI1JMK were amplified by the primer pair *OsSerk2A260c-F*/*Xak1c-R(w/stop)*: 5'-CACCATGGCAATTGGATTTGCATGGTG-3'/5'-TTACCTCGGGCCGGACAGCTCCATTGC-3', *OsSerk2-799F/OsSerk2c-R(w/stop)*:5'-CACCCGGCGGCGTAAA CCTGAAG-3'/5'-TTACCTCGGGCCGGACAGCTCCATTGC-3', *OsSerk2-721F /OsSerk2c-*

R(w/stop): 5’-CACCCGGCGGCGTAAACCTG AAG-3’/5’-TTACCTCGG GCCGGACAGCTCCATTGC-3’, *Xa21-2029F*/*Xa21-3058R*: 5’-CACCCACAAGAGAACTAAAAAGGGAG-3’/5’-TCAGAATTCAAGGCTCCCACC-3’, *Xa3JMK-F*/*Xa3JMK-R(w/stop)*: 5’-CACCATGTATGTAGTGATTAGAAAGAAA-3’/5’-ATCACTGCTGCACAACGCTCACTGT-3’), *OsFLS2JMK-F*/*OsFLS2JMK R(w/stop)*: 5′-CACCATGGAGAGGAATAAATTTGCAAGCA-3′/5′-TCAGTCTTCTCCGACGAGCTT-3′ and *OsBri1JMK-F*/*OsBri1JMK-R(w/stop)*: 5’-CACCATGATAGCCATTGGGAGCAAGCG-3’/5’-CTAATCCTTCTCCTCCTTGGCTTCCC-3’) respectively and were cloned into pENDRTM/D-TOPO® (Invitrogen) to create pENTR-OsSERK2JMK, pENTR-OsSERK2JK, pENTR-OsSERK2TJK, pENTR-XA21JK, pENTR-XA3JMK, pENTR-OsFLS2JMK, and pENTR-OsBRI1JMK. The catalytically inactive variants, pENTR-OsSERK2JMK^{K334E}, pENTR-OsSERK2JK^{D433N} and pENTR-OsSERK2TJK^{D433N} were created by site-directed mutagenesis using pENTR-OsSERK2JMK, pENTR-OsSERK2JK, and pENTR-OsSERK2TJK plasmids, as DNA template and *K334E-1*/-*2*: 5’-GGCTCGTTGGTAGCAGTGGAAAGATTAAAAGAAGAAC-3’ /5’-GTTCTTCTTTTAATCTTTCCACTGCTACCAACGAGCC-3’ or *D433N-1*/-*2*: 5’-CCAAGATCATTCATCGTAATGTCAAAGCTGCAAATATTC-3’/5’-GAATATTTGCAGCTTTGACATTACGATGAATGATCTTGG-3’ as primer pairs. pENTR-OsSERK2JMK, pENTR-OsSERK2JK, pENTR-OsSERK2TJK, pENTR-OsSERK2JMK^{KE}, pENTR-OsSERK2JK^{DN}, pENTR-OsSERK2TJK^{DN}, pENTR-

OsFLS2JMK and the previously constructed vectors pENTR-XA21K668 and pENTR-XA21K668^{KE} [1] were recombined with pB42ADgc [1] to yield the HA fusion protein AD vectors, HA-OsSERK2JMK, HA-OsSERK2JK, HA-OsSERK2TJK, HA-OsSERK2JMK^{KE}, HA-OsSERK2JK^{DN}, HA-OsSERK2TJK^{DN}, HA-OsFLS2JMK, HA-XA21K668 and HA-XA21K668^{KE}. BD vectors, LexA-XA21K668 and its catalytically inactive variant LexA-XA21K668^{K736E} were created as described previously [1]. LexA-XA21K668^{D841N} was created by site-directed mutagenesis using LexA-XA21K668 plasmid as template and *D841N-1/-2*: 5'-CCCTGAACCTGTTGTACACTGTAATATTAAATCAAGCAATG-3'/5'-CATTGCTTGATTTAATATTACAGTGTACAACAGGTTCAGGG-3' as the primer pair. pENTR-XA21JK, pENTR-OsSERK2JMK and pENTR-OsFLS2JMK were then recombined with pLexAgtw [1] to get LexA-XA21JK, LexA-OsSERK2JMK and LexA-OsFLS2JMK. LexA-OsFLS2-JMK was created by site-directed mutagenesis using the LexA-OsFLS2-JMK^{D-N} plasmid as template and OsFLS2D-N-1/-2 5'-CCCCGTCGTGCACTGCAACGTCAAGCCGTCCAACGTC-3'/5'-GACGTTGGACGGCTTGACGTTGCAGTGCACGACGGGG-3' primers. LexA-XA21JK^{D841N} was created by site-directed mutagenesis using the LexA-XA21JK plasmid as template and *D841N-1/-2*: 5'-CCCTGAACCTGTTGTACACTGTAATATTAAATCAAGCAATG-3'/5'-CATTGCTTGATTTAATATTACAGTGTACAACAGGTTCAGGG-3' primers.

For GST- or His-Nus- fusion protein constructions, pENTR-OsSERK21JMK, pENTR-OsSERK2JMK^{K334E}, pENTR-OsSERK2JK and pENTR-OsSERK2JK^{D433N}

were recombined with the gateway compatible vectors pDEST15 (for GST fusion protein) to create the constructs, GST-OsSERK2JMK GST-OsSERK2JMK^{KE} GST-OsSERK2JK and GST-OsSERK2JK^{DN}. The coding cDNA sequence with stop codon of *OsSerk1* encoding OsSERK1JMK, which contains the juxtamembrane and kinase domains (JMK) (amplified by the primer pair *OsSerk1G257c-F*/ *OsSerk1G257c-R(w/stop)*: 5'-CACCATGGGTTTTGCATGGTATCGGCGC-3'/5'-TTATCATCTCGGCCCTGATAGCTCAACCG-3') was cloned into pENDRTM/D-TOPO® (Invitrogen) to create pENTR-OsSERK1JMK. pENTR-OsSERK1JMK was recombined with pDEST15 resulting in the construct for GST-OsSERK1JMK. pENTR-XA21K668^{D841N} was created by site-directed mutagenesis using pENTR-XA21K668 plasmid as template and *D841N-1*/*-2*: 5'-CCCTGAACCTGTTGTACACTGTAATATTAAATCAAGCAATG-3'/5'-CATTGCTTGATTTAATATTACAGTGTACAACAGGTTCAGGG-3' primers. pENTR-XA21K668, pENTR-XA21K668^{K736E} [1], pENTR-XA21K668^{D841N}, pENTR-XA21JK and pENTR-XA21JK^{D841N} were recombined with the gateway compatible vector pET-57-DEST (for 6XHis-Nus- fusion protein) (Novagen) to create the constructs, His-Nus-XA21K668, His-Nus-XA21K668^{KE}, His-Nus-XA21K668^{DN}, His-Nus-XA21JK and His-Nus-XA21JK^{DN}, respectively.

Reference

1. Chen X, Chern M, Canlas PE, Ruan D, Jiang C, et al. (2010) An ATPase promotes autophosphorylation of the pattern recognition receptor XA21 and inhibits XA21-mediated immunity. Proc Natl Acad Sci U S A 107: 8029-8034.

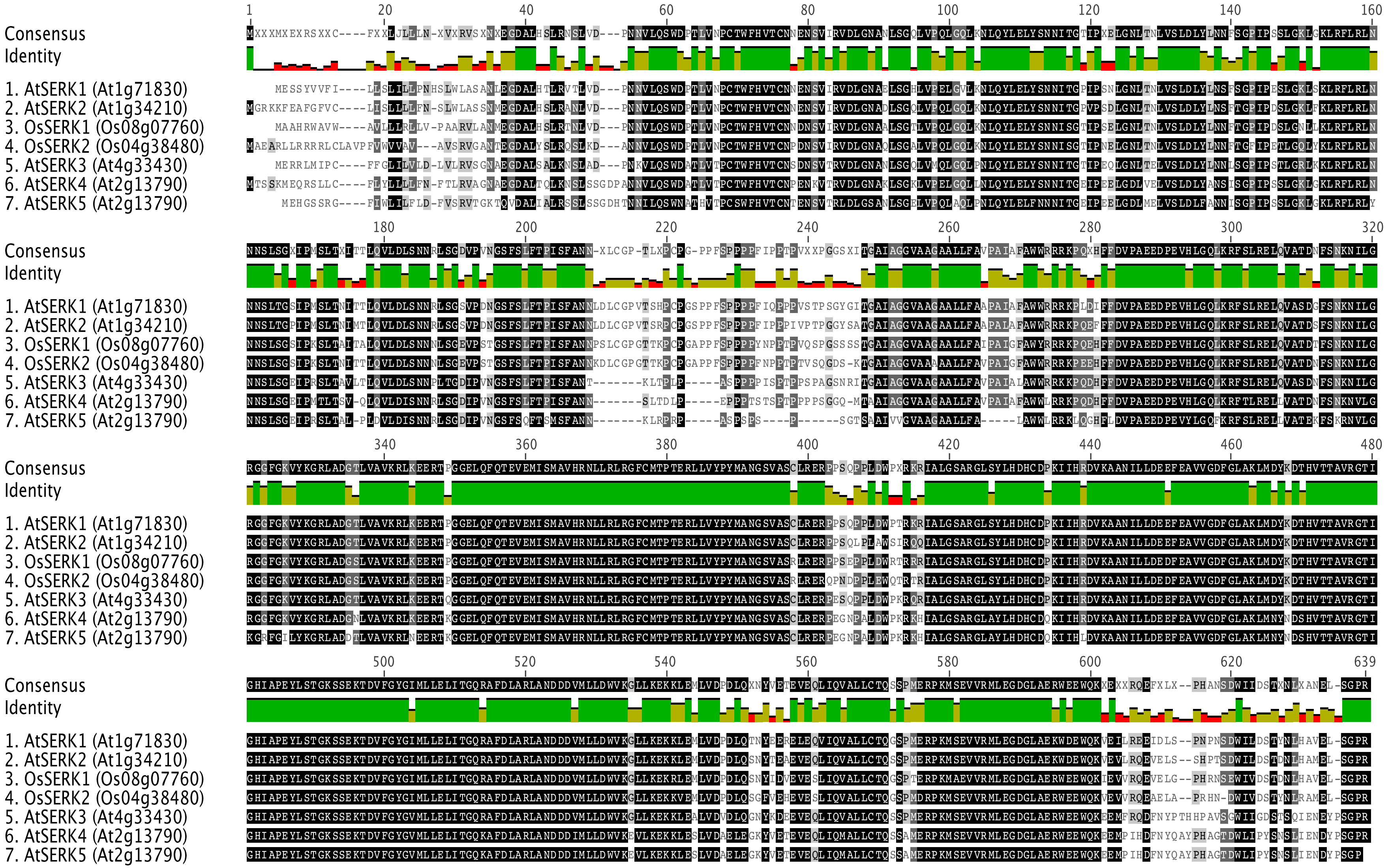

Supplemental Figure 1: Alignment of the five *Arabidopsis* and two rice SERK proteins

Multiple alignments of the five *Arabidopsis* SERKs and two rice SERKs using MUSCLE.

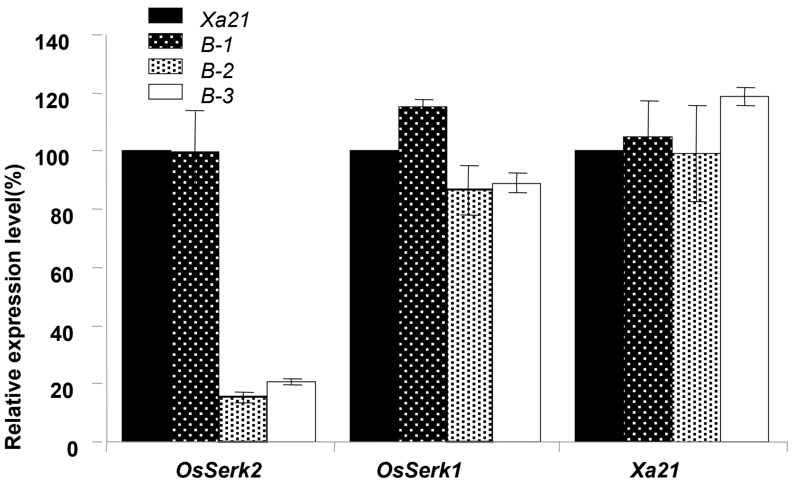

Supplemental Figure 2. Identification of *XOsSerk2Ri* transgenic lines with reduced expression of *OsSerk2*

The relative expression level of *OsSerk1*, *OsSerk2* and *Xa21* was determined by quantitative RT-PCR using RNA extracted from rice leaves of three independently transformed *XOsSerk2Ri* lines (*B-1*, *B-2*, and *B-3*) and *Xa21* transgenic plants. The expression of each gene was normalized to the *actin* reference gene expression level. The expression level of each gene is shown as percentage expression relative to the *Xa21* control plants. Data shown represent average expression level of one out three biological experiments with error bars indicating standard deviation (SD) of three technical replicates.

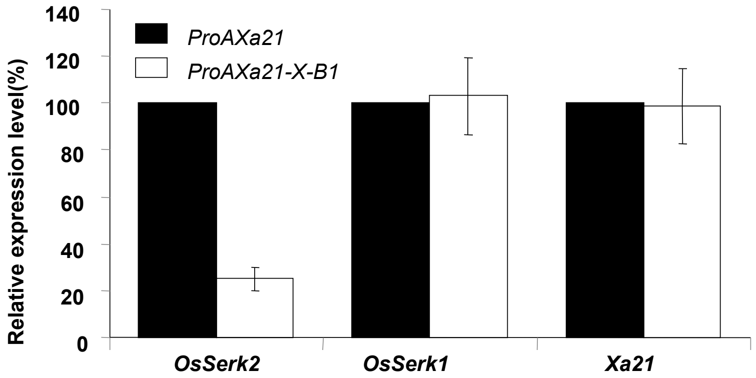

Supplemental Figure 3. Identification of a*ProAXOsSerk2Ri* transgenic line with reduced expression of *OsSerk2*

The relative expression level of *OsSerk1*, *OsSerk2* and *Xa21* was determined by quantitative RT-PCR using RNA extracted from rice leaves of *ProAXOsSerk2Ri* (*ProAXa21/X-B-1*), and *Xa21* transgenic plants. The expression of each gene was normalized to the *actin* reference gene expression level. The expression level of each gene is shown as a percentage expression relative to the *Xa21* control plants. Data shown represent average expression level of one out three biological experiments with error bars indicating SD of three technical replicates.

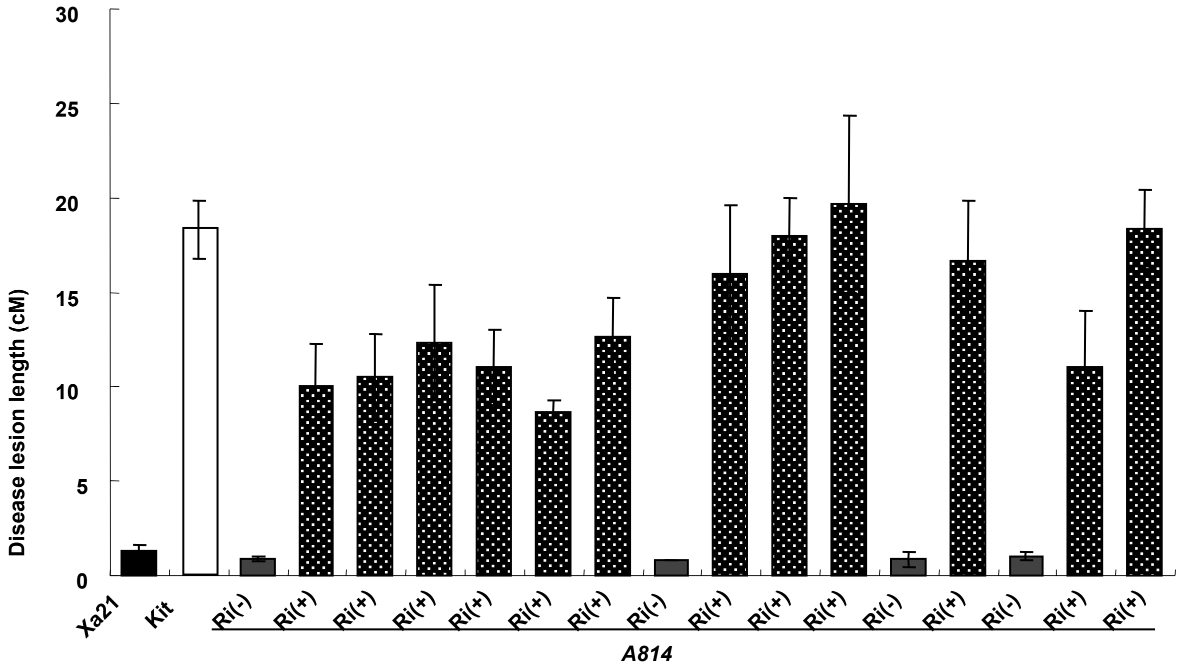

Supplemental Figure 4. Average lesion length in the T1 generation of *XOsSerk2Ri2* plants

Lesion length was measured 14 days post inoculation with *Xoo* strain PXO99AZ. "*Ri(+)*" indicates T1 plants (*A814* derived from *B-1*) carrying the transgene *OsSerk2Ri* whereas "*Ri(-)*" indicates T_1 plants without *OsSerk2Ri* transgene. This experiment was repeated three times with similar results.

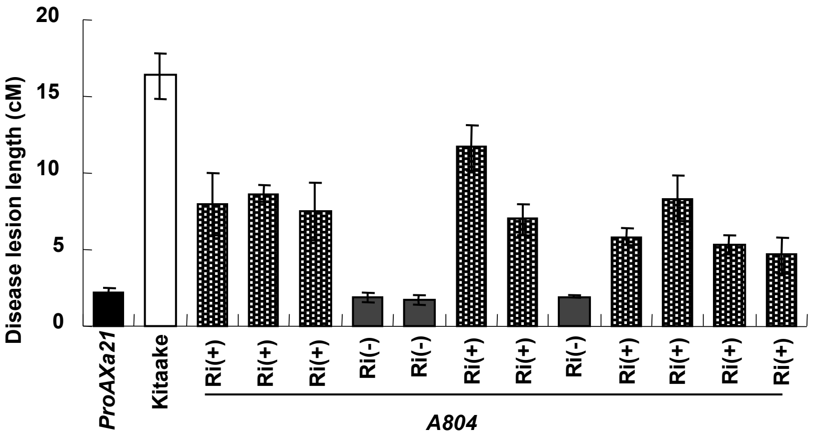

Supplemental Figure 5. Average lesion length in the T1 generation of *ProAXOsSerk2Ri* plants
Disease lesion length was measured 14 days post inoculation with *Xoo* strain PXO99AZ. "*Ri(+)*" indicates T1 plants (*A804* derived from *ProAXa21/X-B-1*) carrying the transgene *OsSerk2Ri* whereas "*Ri(-)*" indicates T1 plants not carrying the *OsSerk2* transgene. This experiment was repeated three times with similar results.

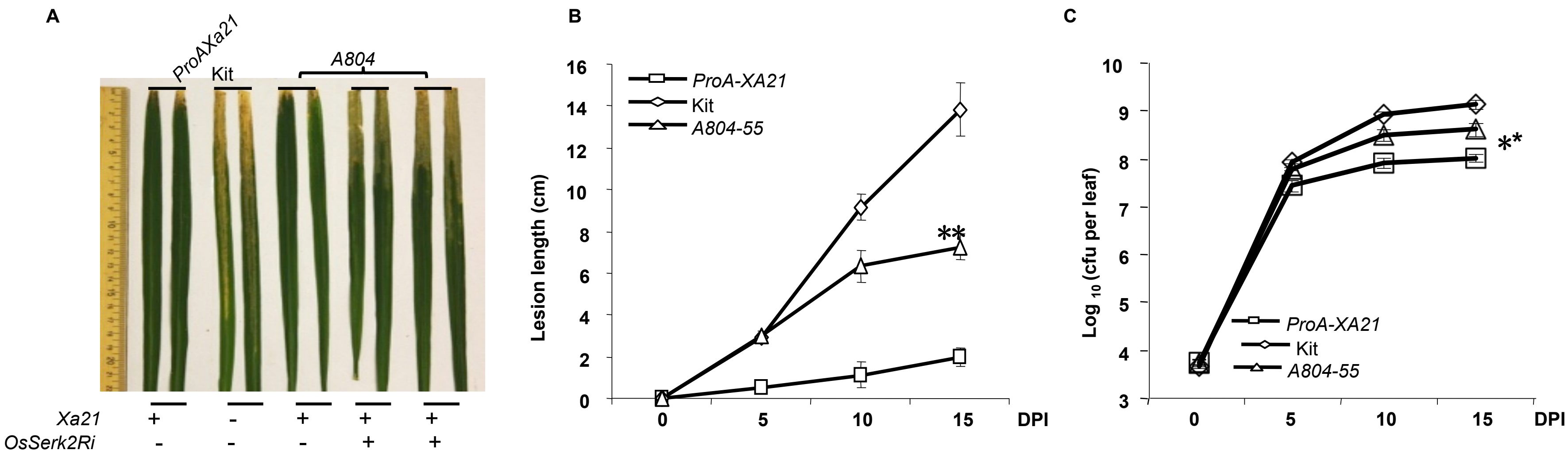

Supplemental Figure 6. Silencing of *OsSerk2* abolishes *XA21*-mediated immunity in *ProAXa21* plants
Six week-old plants of *ProAXOsSerk2Ri1* (*A804*), *ProAXa21* (resistant control) and Kit (susceptible control) were inoculated with *Xoo* PXO99AZ. (A) *A804* plants in the presence of *OsSerk2Ri* develop long water-soaking lesions. Photograph depicts representative symptom development in leaves 14 days post inoculation. "+" and "-" indicate absence or presence of the *Xa21* and *OsSerkRi* transgene, respectively. (B) *ProAXOsSerk2Ri* plants (*A804-55* homozygous for *OsSerkRi*) develop long water-soaking lesions. Lesion length was measured 0, 5, 10, and 15 days post inoculation. Graph shows average lesion length ± SD of at least 21 leaves from 7 independent plants. Statistical significance comparing *A804-55* with *Xa21* plants is indicated by asterisks (**$P \leq 0.05$, ANOVA analysis, Tukey's test). (C) *A804-55* plants display susceptibility to *Xoo* PXO99AZ. Bacterial populations were counted 0, 5, 10, and 15 days post-inoculation. Each data point represents the average ± SD of six leaves from two independent plants. The statistical significance between *A804-55* with *Xa21* plants is indicated by asterisk (**$P \leq 0.05$, ANOVA analysis, Tukey's test). These experiments were repeated at least three times with similar results.

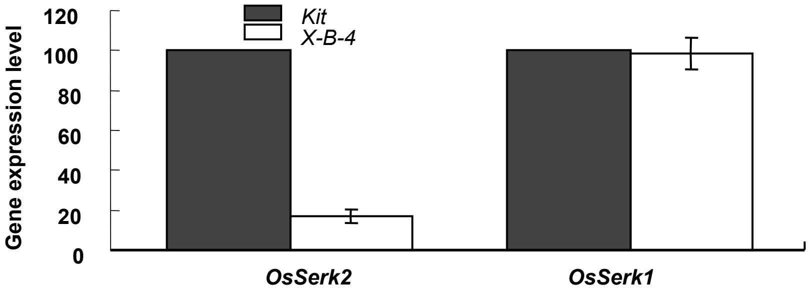

Supplemental Figure 7. Identification of one *OsSerk2Ri* transgenic Kitaake rice line with reduced expression level of *OsSerk2*

The relative expression level of *OsSerk1* and*OsSerk2* and *Xa21* was determined by quantitative RT-PCR using RNA extracted from rice leaves of *Kit-OsSerk2Ri-4* (*X-B-4*) and *Kitaake* plants. The expression of each gene was normalized to the *actin* reference gene expression level. The expression level of each gene is shown as percentage expression relative to the *Kitaake* control plants. Data shown represent average expression level of one out three biological experiments with error bars indicating standard deviation (SD) of three technical replicates.

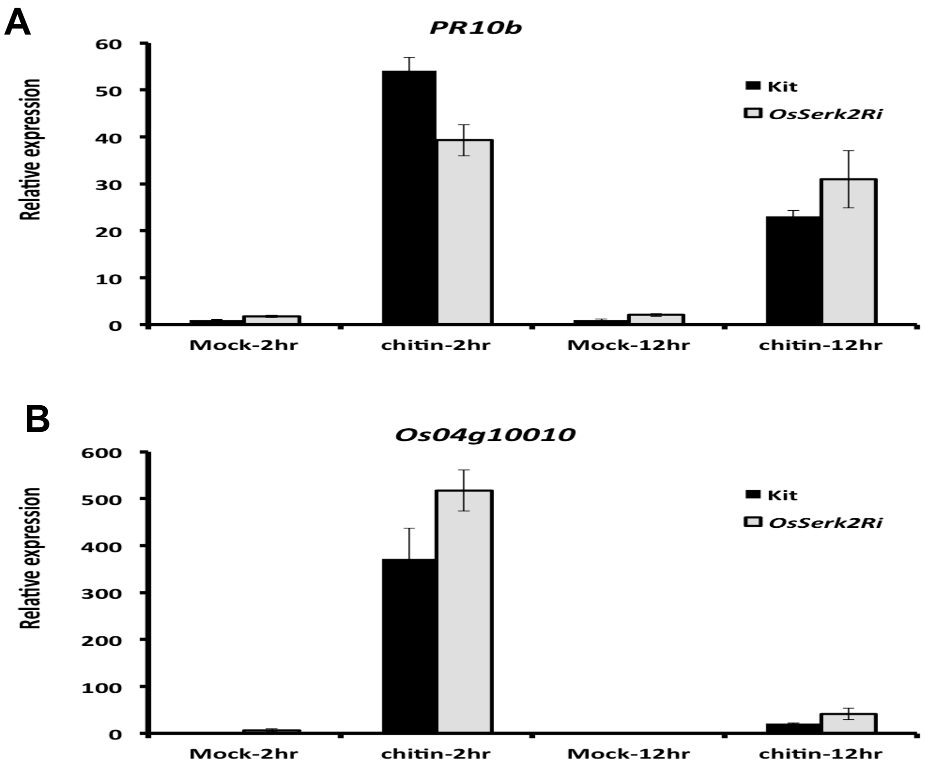

Supplemental Figure 8: Silencing of *OsSERK2* does not interfere with chitin induced defense gene expression in rice

Leaf strips of four-week old Kitaake control or *OsSerk2Ri*(X-B-4-2) plants were treated or not with 50 ug/ml of chitin for 2 or 12 hours. Expression levels of the two defense marker genes *PR10b* (A) and *Os04g10010* (B) were measured by quantitative RT-PCR. Expression levels for each gene were normalized to actin reference gene expression. Data shown is normalized to Kitaake mock control at 2h or 12h, respectively. Bars depict average expression level ± standard error of two biological replicates with two technical replicates each. This experiment was repeated three times with similar results.

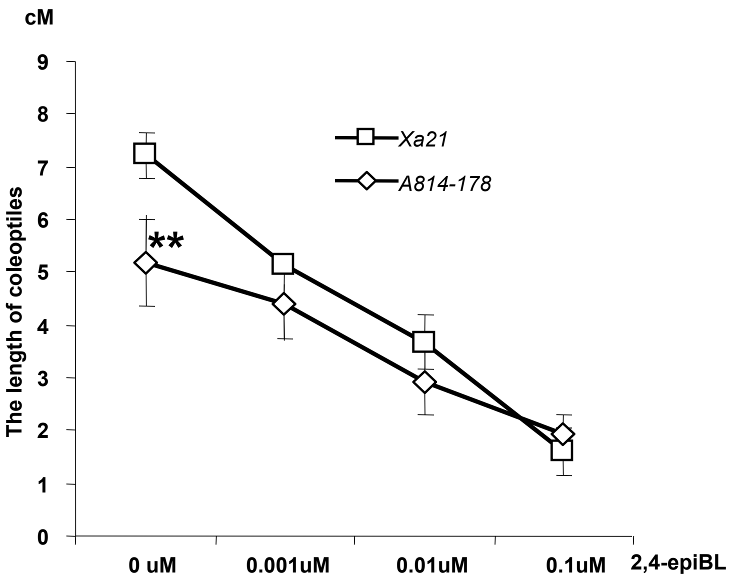

Supplemental Figure 9. Silencing of *OsSerk2* impairs BR signaling

The "X" axis represents different concentrations of 24-epiBL (Sigma). The "Y" axis stands for the length of coleoptiles. Transgenic plants of *Xa21-OsSerk2Ri-2* (*A814-178*) with stable reduced expression of *OsSerk2* were used for analyses. *Xa21* plants were used for control. The asterisks indicate a significant difference between *OsSerk2* silenced and control lines (**$P \leq 0.05$, ANOVA analysis, Tukey's test). This experiment was repeated three times with similar results.

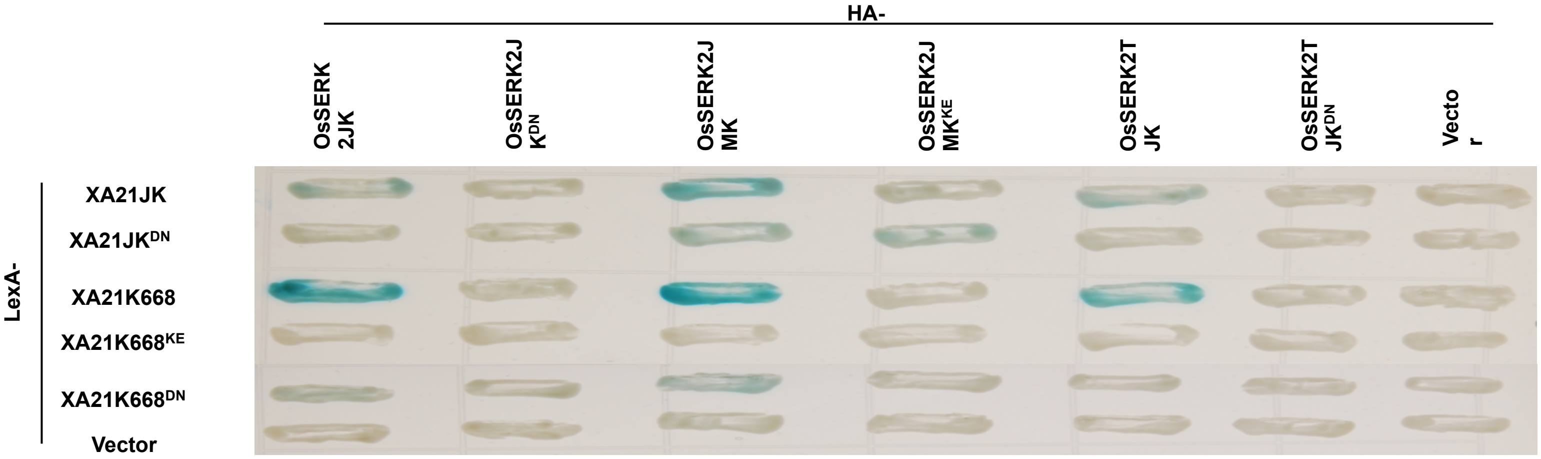

Supplemental Figure 10. Determination of the interaction between catalytically inactive OsSKERK2 and XA21 mutant variants

HA-OsSERK2JK (OsSERK2JK), HA-OsSERK2JMK (OsSERK2JMK), HA-OsSERK2TJK (OsSERK2TJK), and their catalytically inactive variants,HA-OsSERK2JK^{D433N} (OsSERK2JK^{DN}), HA-OsSERK2JMK^{D433N} (OsSERK2JMK^{DN}), HA-OsSERK2TJK^{D433N} (OsSERK2TJK^{DN}) and empty vector pB42AD were co-transformed with LexA-XA21JK (XA21JK), LexA-XA21K668 (XA21K668) and their catalytically inactive variants, LexA-XA21JK^{D841N} (XA21JK^{DN}), LexA-XA21K668^{K736E} (XA21K668^{KE}), LexA-XA21K668^{D841N} (XA21K668^{DN}) and the empty vector pLexA, respectively. The Matchmaker LexA two-hybrid system (Clontech) was used for the yeast two-hybrid experiments. The blue color indicates nuclear interaction between the two co-expressed proteins. This experiment was three times with similar result.

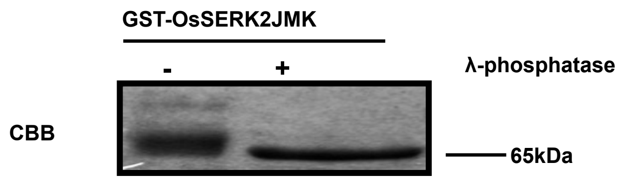

Supplemental Figure 11. GST-OsSERK2 is hyperphosphorylated when heterologously expressed in *E.coli*
GST-fused OsSERK2JMK proteins (GST-OsSERK2JMK) were incubated with 1 X Protein MetalloPhosphatases (PMP) buffer in the presence (+) or absences (-) of Lambda Protein Phosphatase (1000U) for 1 hour at 30^0C. Proteins were separated by SDS-PAGE and stained with CBB.

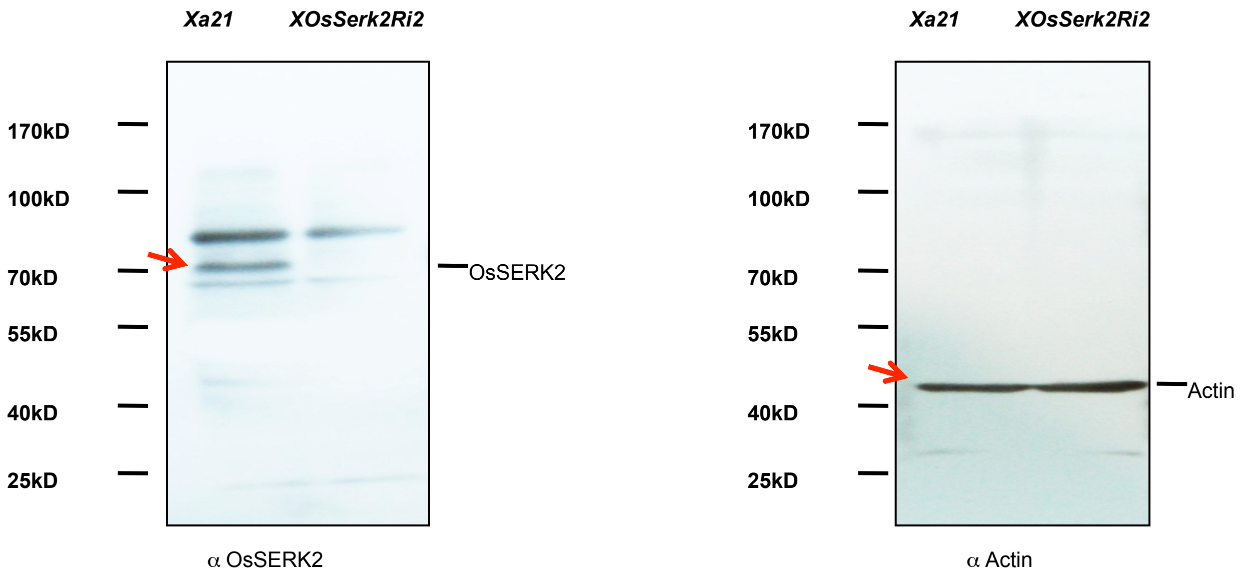

Supplemental Figure 12: Validation of anti-OsSERK2 antibody specificity on total rice protein extracts

The anti-OsSERK2 antibody recognizes a specific protein of approximately 70kDa in size in total rice protein extracts from *Xa21* plants but not from *OsSerk2*-silenced *Xa21* plants (*XOsSerkRi2*) (left panel). 75ug of total protein for each genotype were separated by SDS-PAGE gel electrophoresis and subjected to immuno-blot analysis with anti-OsSERK2 (left panel) or anti-Actin (right panel) antibody as loading control, respectively. Full membranes for each immuno-bot are shown. The arrows indicate the cropped bands cropped that are shown in Figure 7B. This experiment was repeated three independent times with similar results.

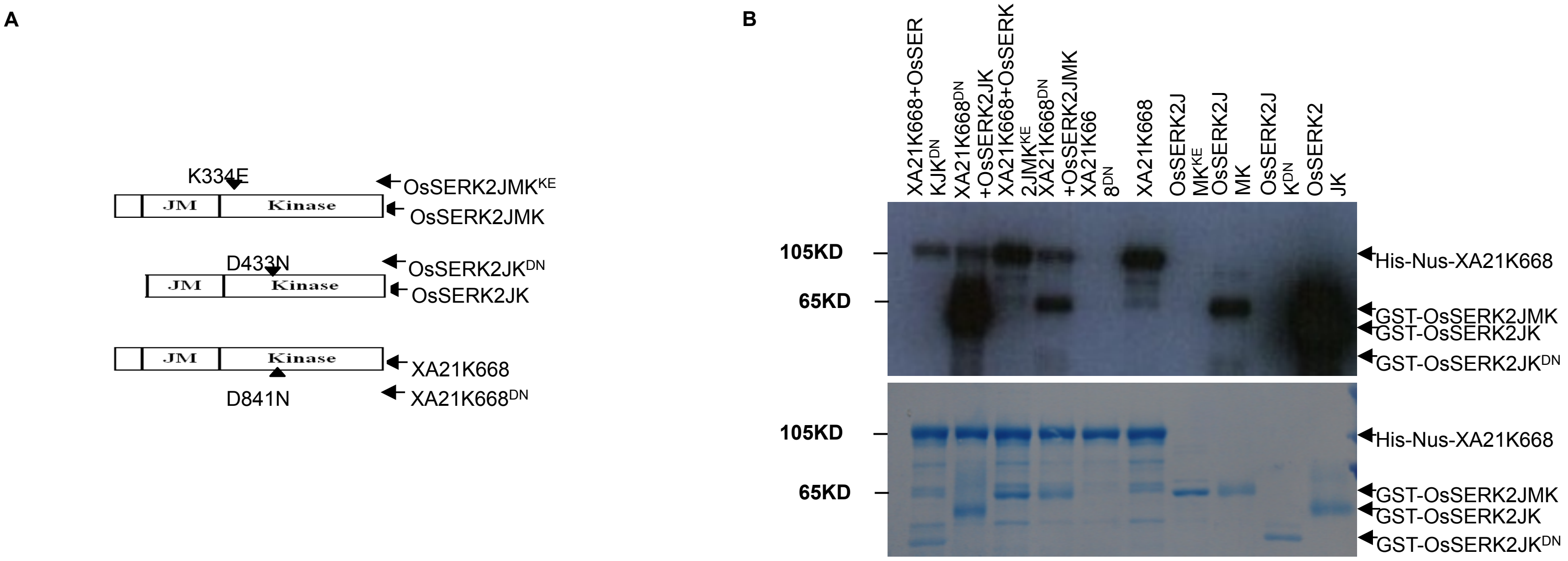

Supplemental Figure 13. OsSERK2JK and OsSERK2JMK transphosphorylates XA21K668 *in vitro* but not vice versa.

(A) Depiction of protein domain architecture usedfor trans-phosphorylation assays. OsSERK2JMK, XA21K668 and their respective kinase inactive variants, OsSERK2JMK^{K334E} (OsSERK2JMK^{KE}), and XA21K668^{D841N} (XA21K668^{DN}) proteins contain part of the TM domain, full JM and kinase domain. OsSERK2JK and its kinase catalytically inactive variant, OsSERK2JK^{D433N} (OsSERK2JK^{DN}), contain full JM and kinase domains but lack part of the TM domain. (B) *In vitro* trans-phosphorylation assay between OsSERK2JK and XA21K668. The assay was performed by incubating GST-OsSERK2JMK (abbreviated as OsSERK2JMK), GST- OsSERK2JMK^{K334E} (abbreviated as OsSERK2JMK^{KE}), GST-OsSERK2JK (abbreviated as OsSERK2JK) and GST-OsSERK2JK^{D433N} (abbreviated as OsSERK2JK^{DN}) in presence or absence of His-Nus-XA21K668 (abbreviated as XA21JK) or His-Nus-XA21K668^{D841N} (abbreviated as XA21JK^{DN}) using radioactive labeled [^{32}P]-γ-ATP. Proteins were separated by SDS/PAGE and analyzed by autoradiography in the top panel and the protein loading control is shown by CBB in lower panel. This experiment was repeated twice with similar results.ç√ç

297-R.ELQVATDNFSNK.N-310 T303

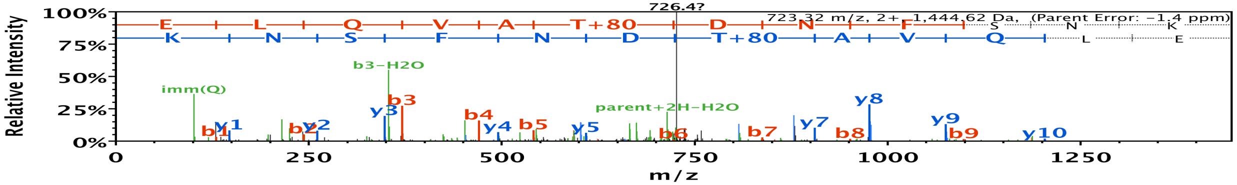

324-R.LADGSLVAVK.R-335 S329

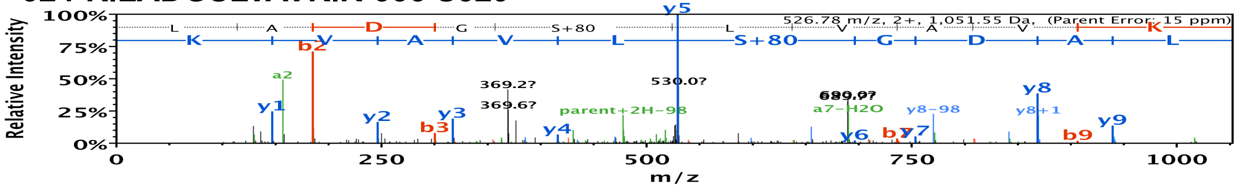

376-R.LLVYPYMANGpSVASR.L-392 S387

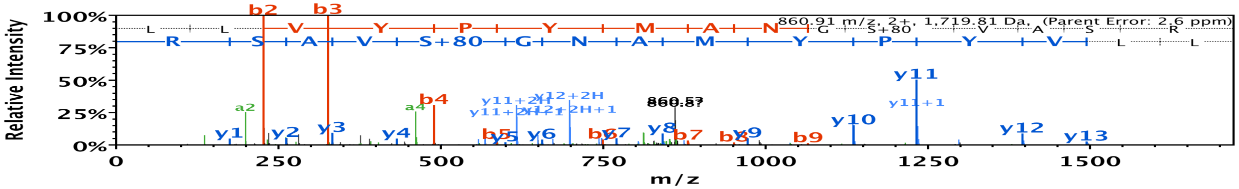

461-K.DTHVpTTAVR.G-471 T466

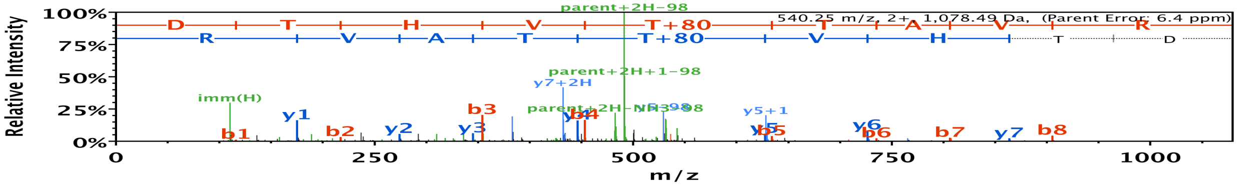

461-K.DTHVTpTAVR.G-471 T467

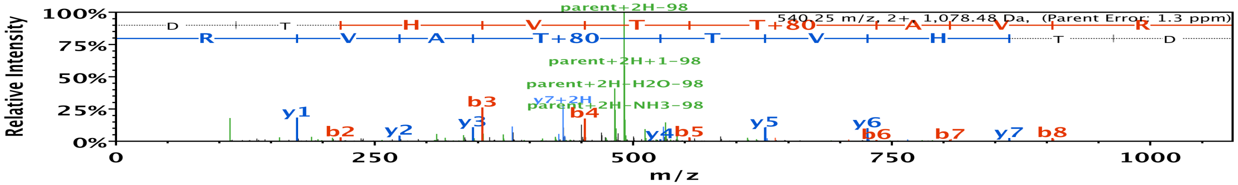

470-R.GpTIGHIAPEYLSTGK.S-486 T472

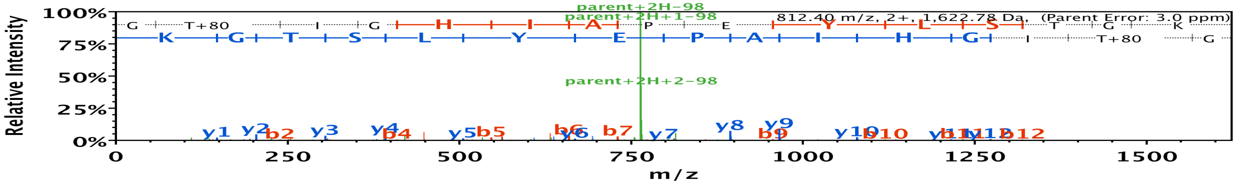

607-R.HNDWIVDpSTYNLR.A-621 S615

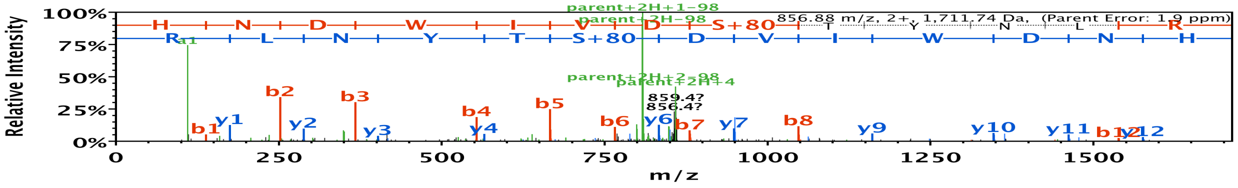

620-R.AMELpSGPR.--628 S625

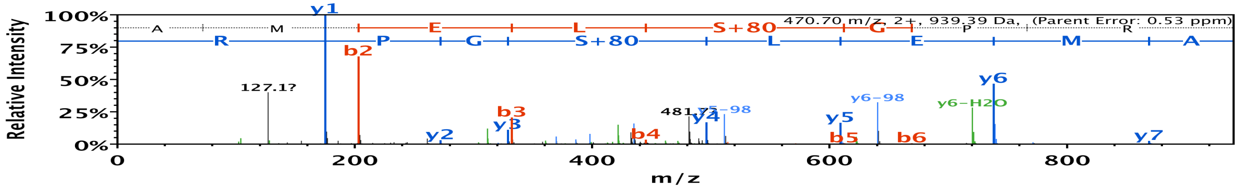

340-R.TPGGELQFQpTEVEMIpSMAVHR.N-362 T350, S356

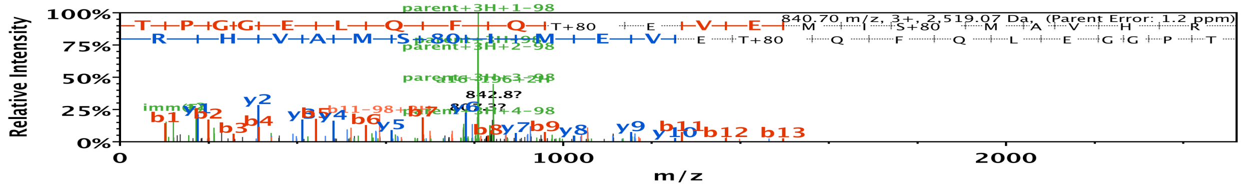

456-K.LMDYKDpTHVpTTAVR.G-471 T463, T466

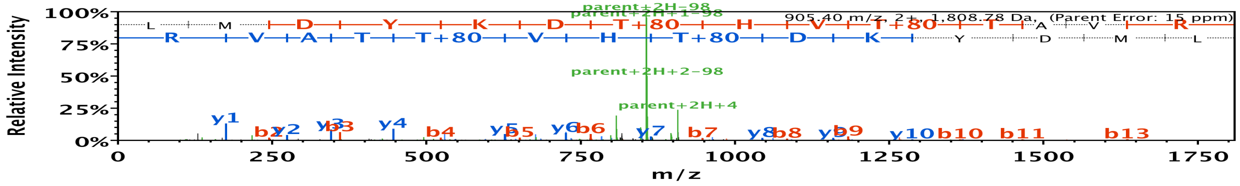

607-R.HNDWIVDpSpTYNLR.A-621 S615, T616

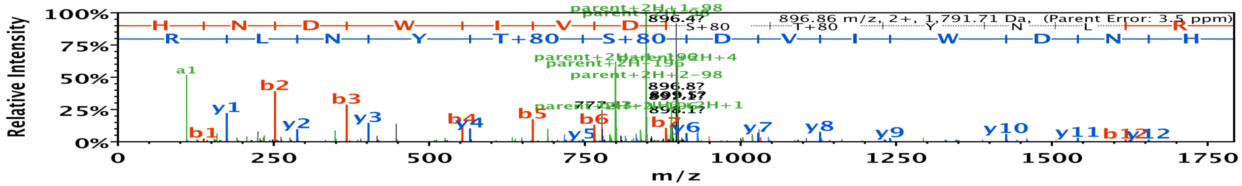

Supplemental Figure 14. MS2 spectra for all peptides given in Table 1
The complete MSMS analyses can be found in Supplemental Data 1.

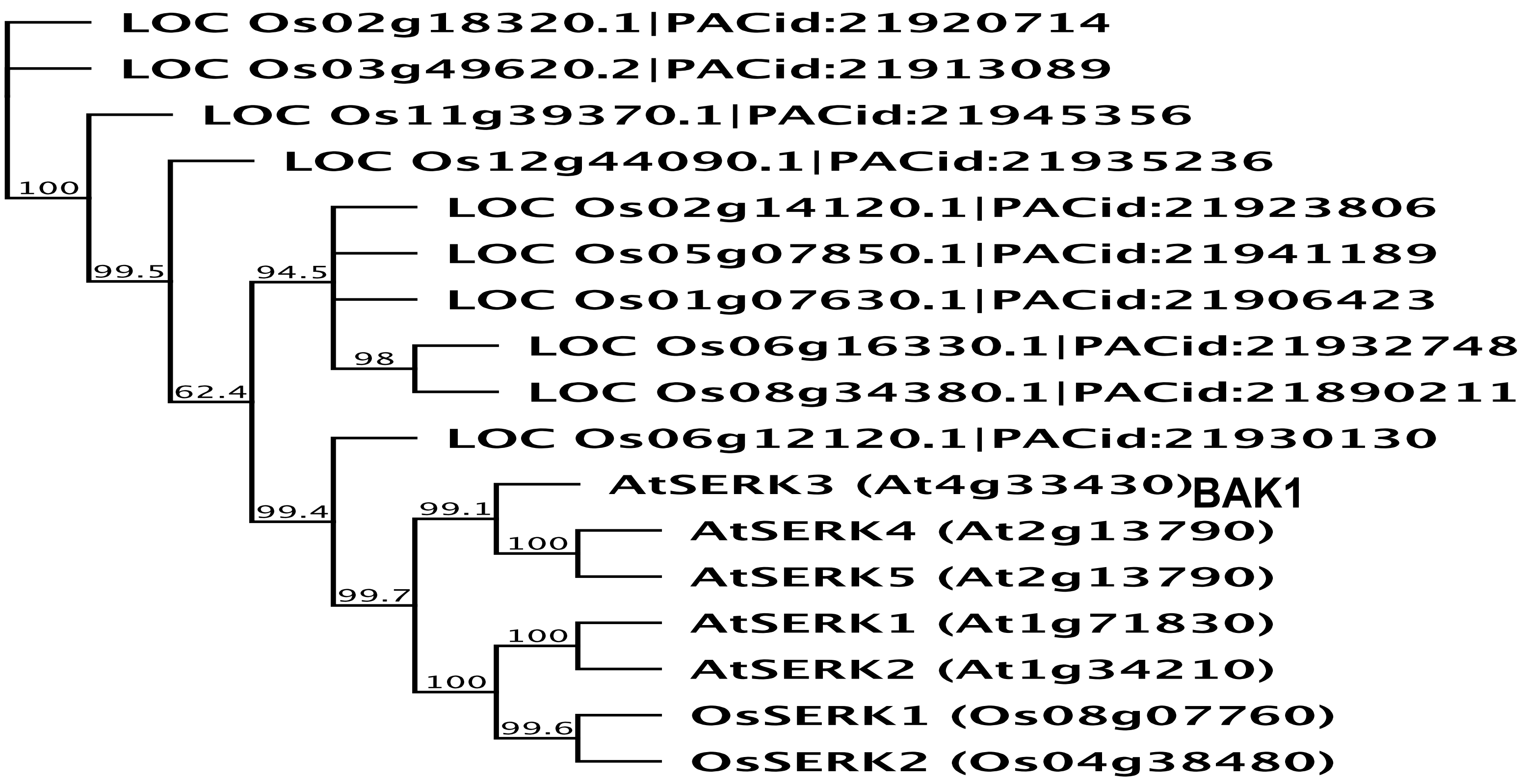

Supplemental Figure 15A. Phylogenetic analysis was performed on the two rice, five *Arabidospsis* SERK proteins and its next 10 closest rice homologs as determined by blastp.
Rice SERK1 and SERK2 were grouped with their five *Arabidopsis* counterparts. Full-length amino acid sequences of all SERK proteins and their ten closest rice homologs were analyzed using Geneious Tree builder. The phylogenetic tree was generated using a bootstrap neighbor-joining tree applying 1,000 replicates. For all SERK proteins identifiers are given in brackets.

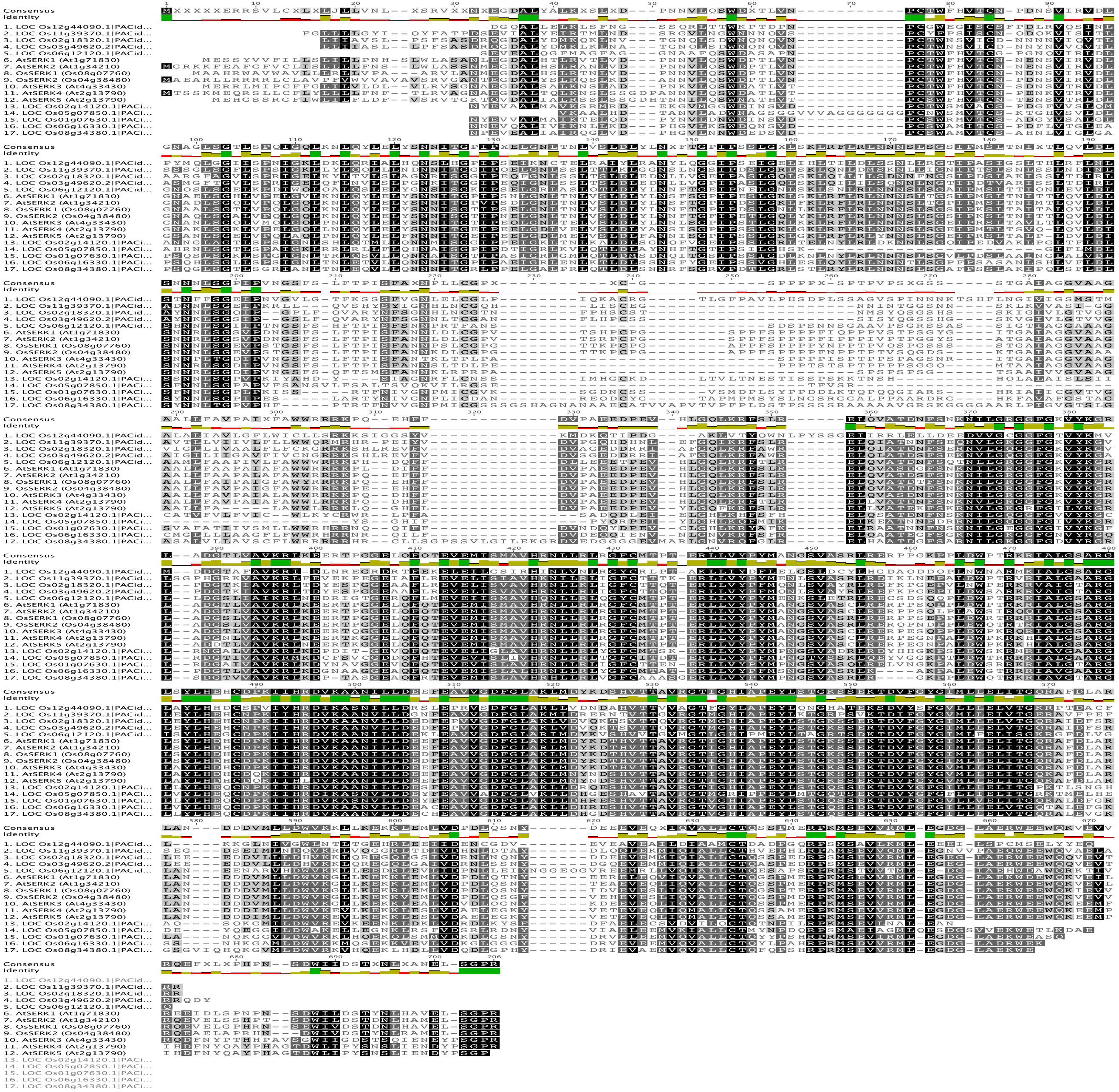

Supplemental Figure 15B. Multiple alignments of the five *Arabidopsis* SERKs, two rice SERKs and their then closest rice homologs using Muscle.

Table. S1

Lines	Expression of *g07760*	Total plants	Genotypes		Disease resistance	
			Hyg (+)	Hyg (-)	Resistance	Susceptibility
Xa21	100%	10	0	10	10	0
XOsSerk1Ri-1	17.4%±4.7%	11	9	2	11	0
XOsSerk1Ri-2	17.0%±5.2%	14	10	4	14	0
XOsSerk1Ri-3	17.0%±3.6%	7	6	1	7	0
XOsSerk1Ri-4	10%±1.6%	14	11	3	14	0
XOsSerk1Ri-6	113%±2.1%	9	4	5	9	0
Kit		10	0	10	0	10
ProAXa21	100%	10	0	10	10	0
ProAXOsSerk1Ri-1	13.5%±2.6%	19	13	3	19	0
ProAXOsSerk1Ri-2	116.8%±1.4%	16	12	4	16	0
ProAXOsSerk1Ri-3	11.5%±0.4%	16	4	12	16	0
Kit		10	0	10	0	10
